# Aging in the Digital Age: Using Technology to Increase the Reach of the Clinician Expert and Close the Gap Between Health Span and Life Span

**DOI:** 10.3389/fdgth.2021.755008

**Published:** 2021-11-19

**Authors:** Joyce Gomes-Osman, Javier Solana-Sánchéz, Emily Rogers, Gabriele Cattaneo, William Souillard-Mandar, David Bates, Enrique J. Gomez, Josep M. Tormos-Muñoz, David Bartrés-Faz, Álvaro Pascual-Leone

**Affiliations:** ^1^Department of Neurology, University of Miami Miller School of Medicine, Miami, FL, United States; ^2^Linus Health, Waltham, MA, United States; ^3^Brain Health and Neurorehabilitation Institute, Institut Universitari de Neurorehabilitació Adscrit a la Universitat Autònoma de Barcelona (UAB), Badalona, Spain; ^4^Departament de Medicina, Facultat de Medicina i Ciències de la Salut i Institut de Neurociències, Universitat de Barcelona, Barcelona, Spain; ^5^Center for Biomedical Technology, Biomedical Engineering and Telemedicine Centre, Escuela Técnica Superior de Ingenieros (ETSI) Telecomunicación, Universidad Politécnica de Madrid, Madrid, Spain; ^6^Centro de Investigación Biomédica en Red, Biomateriales y Nanomedicina, Madrid, Spain; ^7^Department of Neurology, Harvard Medical School, Boston, MA, United States; ^8^Hinda and Arthur Marcus Institute for Aging Research and Deanna and Sidney Wolk Center for Memory Health, Hebrew SeniorLife, Boston, MA, United States

**Keywords:** digital health, cognitive impairment, dementia, early detection, technology

## Abstract

Age-related cognitive impairment (ARCI) has a profound impact on individuals, families, health care systems, and societies at large. Evidence suggests that ARCI is the consequence of underlying brain pathology. Therefore, efforts to minimize the impact of ARCI and thus closing the gap between health span and life span, which has widened in recent years, requires early detection and timely deployment of targeted, personalized interventions. Access to clinical experts is limited and technology screening and assessment methods are thus appealing. However, as traditionally implemented patients were deprived of the benefit of personalized connection with a clinician, which is particularly critical for the prescription and to ensure the adherence to and ultimate success of therapeutic interventions. We present the concept of Intelligent Technology Therapy Assistant (ITA) as a scalable solution that increases the reach of clinical experts while sustaining the personal connection between each patient and their clinician. We illustrate ITA with the “Guttman Neuro Personal Trainer”^®^, a tele-rehabilitation platform that provides neuropsychological evaluation and care, and the Barcelona Brain Health Initiative (BBHI) multimodal intervention coaching app, a mobile-based platform that provides lifestyle coaching support in domains related to brain health. In addition, we discuss the translation of these models to a large-scale enterprise with Linus Health. To this end, we conclude with a discussion of challenges and opportunities to move the field forward.

## Introduction

### The Widening Gap Between Health Span and Lifespan

By 2050 the global older adult population will double from what it was in 2015, from 8.5 to 16.7% of the total world population. The increase in human longevity has unfortunately not been accompanied by a comparable increase in healthy longevity. In the last 20 years, global life expectancy increased by nearly seven years (from 66.8 to 73.4), but approximately only five are estimated to be healthy years mostly due to decreased mortality and increased living standards. Notably, only 0.36 year (about 4 months) could be attributed to reduced disability (1).

Alzheimer's Disease and related dementias (ADRD) are a significant cause of disability in aging, and currently the 6th leading cause of mortality in the older population (2). Maintaining cognitive function is a key predictor of greater quality of life and independence in aging (3). The implication of these findings is that successful strategies to improve healthy longevity are critically needed to ensure that additional years gained will not be spent under the burden of serious illness.

### Current Practices, Their Strengths, and Limitations

AD is the most common type of dementia (4), characterized by brain atrophy, synaptic dysfunction, histologic findings of neuritic plaques containing Aβ accumulation and neurofibrillary tangles containing phosphorylated tau, assessed through functional magnetic resonance imaging (MRI), cerebrospinal fluid measurements, positron emission tomography (PET) (5), and more recently with the introduction of a novel blood test (6). In addition to neuropathology, other factors such as genetic predisposition, familial history, environmental exposures, and importantly, who the individual is and how they have lived their life, are all factors to either confer risk or protection (7–9).

AD has a long preclinical stage, wherein biomarkers of neuropathology can be detected decades before any clinically detectable cognitive impairment (5). The combination of this long preclinical stage with multifactorial risk factors that interact with one another in ways that we only partially understand, has contributed to the current lack of effective disease-modifying treatments for dementia. And while these advances in AD neuropathology knowledge, biomarkers, and risk factors are encouraging and point to a brighter future, at the present time, clinically AD is not diagnosed prior to symptom onset (10).

Dementia diagnosis is mostly performed in primary care settings (with specialist referrals when appropriate) and relying largely on medical history and screening tools such as the Mini-Mental State Exam (developed in 1975) and Montreal Cognitive Assessment (MoCA, developed in 1996), and followed up using these metrics on a yearly basis. Additional workup includes comprehensive neuropsychological assessments, blood tests, and neuroimaging. Despite the existence of cognitive screening tools with higher sensitivity and specificity than those widely used in the clinical setting, a systematic review performed in 2020 by the US Preventive Services Task Force found no established benefit in conducting universal cognitive screening for adults older than 65 as there was insufficient evidence that such information could change clinical decisions, management or planning by clinicians, caregivers or individuals (11).

Compelling evidence demonstrates that lifestyle modification strategies such as engagement in physical activity, promotion of healthy sleep, healthy diet, engagement in cognitively stimulating activities, and social engagement have been independently associated with measurable delays in the development of dementia (8, 9). Lifestyle modification strategies are widely recommended, but rarely systematically applied in the clinical setting. Identified barriers are the limited bandwidth of time and resources for clinicians to guide such interventions, and practical aspects such as limited or no direct insurance coverage. Furthermore, evidence suggests that rather than receiving expert advice, contemporary approaches grounded by science of behavior change are most effective at promoting behavior change (12–14), but require time and techniques that are practically challenging to implement in most clinical settings.

Another key barrier to progress is the limitation in the availability of neuropsychological assessments that are sensitive to more subtle changes, or change from interventions, which typically span from weeks to months. Furthermore, the interpretation of such tests is typically done by comparisons to group means rather than individual elements of one's own performance, which could be more sensitive to monitor performance changes that will lead to clinical symptoms.

Addressing these barriers is of utmost importance, since it is estimated that the US alone spent $305 billion in healthcare costs for AD treatment in 2020 and is expected to grow to $1 trillion by 2050 at the current demographic trend and course of treatment (15). Delaying the onset of dementia due to AD for 5 years would result in over 40% lower prevalence and 40% lower cost in 2050 (16). For the individual patient it would translate into 2.7 additional life years and a total value of over $500,000 per person.

## The Intelligent Technology Therapy Assistant

High-quality medical care requires responsibility not only based on knowledge and clinical skills, but also in managing a vast amount of information related to patient care (17). Decision Support Systems (DSS) are defined as information systems that aids in decision-making activities requiring judgement, determination, and a sequence of actions. Thanks to advances in health assessment technologies, and importantly, data mining techniques, machine learning, and other artificial intelligence (AI), DSS have been widely used in clinical practice for the last few years and increasing efficiency and extracting knowledge from large amounts of data from information systems (18).

**Figure 1:**
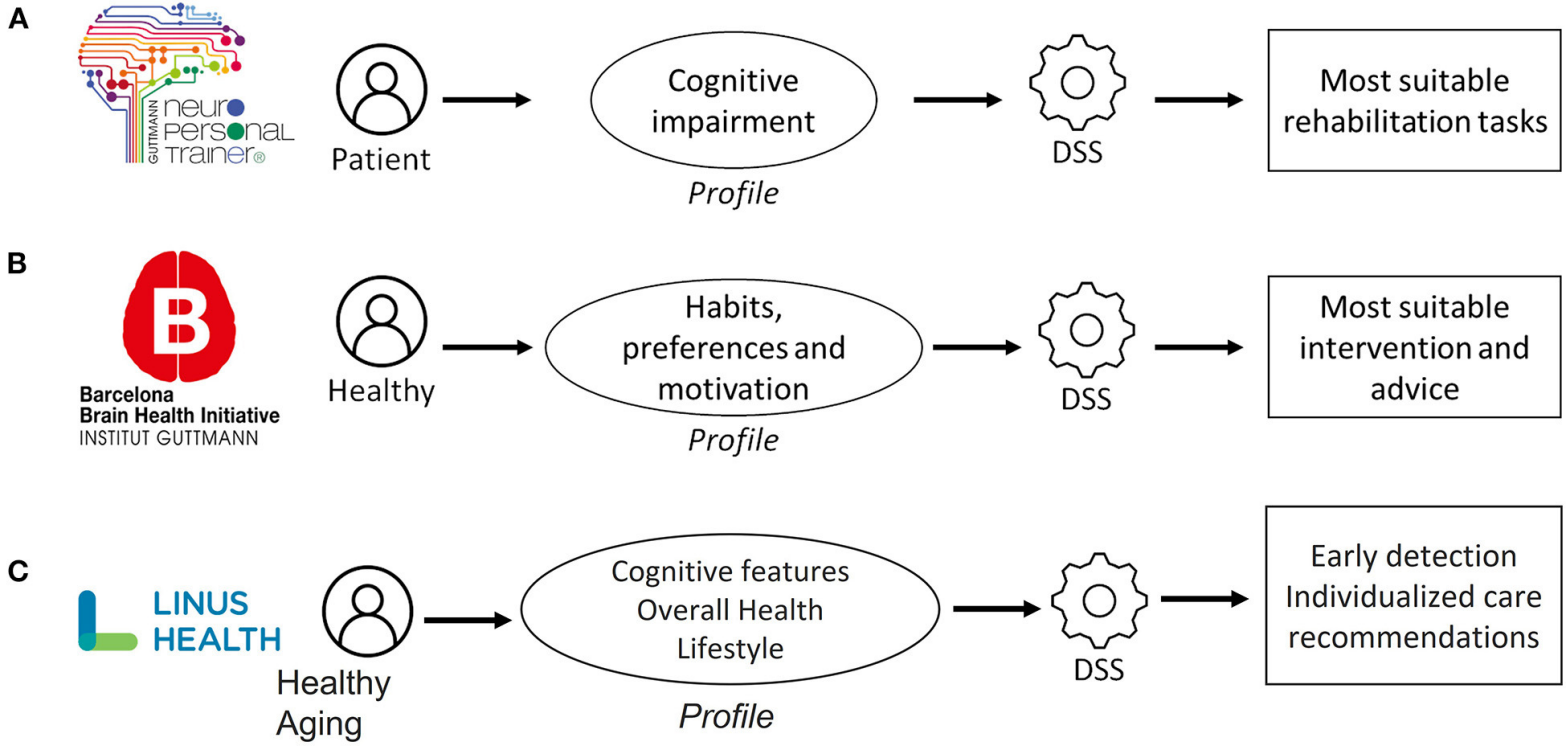
Specific frameworks that illustrate the applied use of digital technologies to expand the reach of the clinician, while sustaining the personal connection between each patient and their clinician. **(A)** Intelligent technology therapy assistant (ITA) “Guttman Neuro Personal Trainer”^®^ **(B)** Barcelona brain health initiative (BBHI) stage 3 coaching app; **(C)** linus health.

In the context of cognitive rehabilitation and training, the Institut Guttmann started to develop in 2008 a tele-rehabilitation platform for treating patients with Brain Injury (19). This web-based platform was designed to allow therapists and neuropsychologists to asynchronously schedule and monitor rehabilitation sessions consisting of computerized tasks. In 2014, the initial platform evolved into the “Guttmann Neuro Personal Trainer”® platform (Figure 1A), adding some new features and improving functionalities, including a DSS tool, named Intelligent Therapy Assistant (ITA, Figure 1A), an algorithm which automatically selected, configured and scheduled rehabilitation tasks for patients with cognitive impairment (20).

At that moment, 1,210 patients had already completed treatment with GNPT. The database included 44,989 rehabilitation sessions and a total of 286,870 executed tasks. Thanks to this available information, the ITA applied data mining techniques to cluster the patients according to their cognitive impairment profile. Then, it rated every rehabilitation task available in the system, based on its cognitive structure and the clinical impact of executions done by similar patients, and also looked for those tasks that were executed for the patients who had better improved their cognitive functioning after the treatment. Finally, it determined the most suitable degree of difficulty configuration, depending on the impairment of the patient and his/her evolution during the treatment.

The validation of the ITA included results from 582 patients, managed by 28 different therapists during 18 months, which meant 20,127 rehabilitation sessions automatically scheduled with 92,813 executed tasks. The obtained results revealed that the rehabilitation treatment proposed by the ITA was as effective as the one performed manually by therapists, revealing it as a new powerful support tool for professionals.

Thanks to the GNPT together with the ITA functionality, the ratio of patients treated simultaneously by neuropsychologists has increased up to 10 times, compared to the traditional face-to-face neuropsychological rehabilitation, since the approximate needed time to schedule 10 sessions for a patient is 5 min. After the validation study was done, the ITA has been successfully used in GNPT for 10 years. Thanks in part to the ITA, GNPT has exponentially grown since 2008, being implemented in more than 400 clinical centers worldwide so far. Looking at the database, +1,400 professionals have used the platform for delivering rehabilitation service to almost 16,000 patients. This means almost 500,000 executed sessions, with more than 22 million executed tasks.

### Barcelona Brain Health Initiative (BBHI) Stage 3 Coaching App

The BBHI (21) is an ongoing prospective longitudinal study focused on identifying determinants of brain health. The main objectives of the initiative are: (i) to characterize through online questionnaires lifestyle, cognitive, behavioral and environmental markers related to a given individual's cognitive and mental functions in middle to old age (Stage 1), (ii) to assess the biological determinants predictive of maintenance of brain health in a subcohort of 1,000 participants (Stage 2), and (iii) to evaluate the impact of a controlled multidomain lifestyle intervention on improving and maintaining brain health through an ICT-based solution (Stage 3, Figure 1B).

In this last stage, a mHealth platform has been designed and developed with the purpose of helping people to improve and monitor their healthy habits, facilitating the delivery of health coaching strategies. This platform consists of two main components: (i) a web portal for the coach that supervises the intervention program and (ii) a mobile application where users receive personalized recommendations to help them to improve their habits in different domains related to brain health: cognition, physical activity, sleep, socialization, vital plan, nutrition, and general health.

The arrival of the COVID-19 pandemic had a great impact on every in-person research activity related to projects like BBHI Stage 3. The crisis was turned into an opportunity by the implementation of a DSS. Thus, an initial profile for the person is created by taking the following input information: (i) current healthy habits profile, (ii) a set of personal preferences, and (iii) motivational aspects to determine which domains the user would be most interested in. This profile would let the system determine the most suitable intervention plan and personalized advice that the user will receive through the app.

Prior to the intervention pilot, an initial validation was done to assess feasibility and usability, including 20 participants during 3 weeks from very diverse territories of Spain, who have been followed and monitored by a single professional. The high score obtained for the usability assessment, plus the adherence to the intervention proposed, showed us that this kind of technology-based intervention is feasible.

### Linus Health

Ultimately, true transformative impact requires scalable ventures with global reach potential. Linus Health is such a venture, an example of leveraging cutting edge digital assessment tools and advanced analytics to develop individualized care recommendations for general and brain health that are data-driven, reproducible and objective. Linus Health seeks measure cognition through assessments, understand patient condition through sophisticated models, and intervene through individualized clinical decision support and care recommendations that are supported by AI (Figure 1C). This framework empowers the clinician and puts the individual at the center of the care continuum. As recommendations are deployed, this information is used to further refine existing models, and apply such learnings to help other individuals.

One example is DCTclock^TM^ (22, 23), a digital version of the Clock Drawing Test, used for over 50 years to screen for cognitive impairment. Participants are asked to draw a pair of clocks, and DCTclock captures and automatically analyzes the assessment using machine learning algorithms, thus reducing bias from subjective judgment. Additionally, it not only captures the final clock drawing score, but also the processes involved, organized in a host of other spatial and temporal metrics that give highly detailed data on a patient's performance. The automated scoring models have significantly outperformed traditional clock drawing test, producing (area under the curve) AUC scores ranging from 0.89 to 0.93 for screening, compared against published AUC scores from existing clinician scoring systems of 0.66–0.79. DCTclock has also been shown to outperform traditional neuropsychological tests like the MMSE in detecting early cognitive impairment and demonstrated the ability to characterize individuals along the AD trajectory (23). In a push to detect the earliest cognitive changes associated with AD, two validation studies of DCTclock on patients with preclinical AD showed significant association of DCTclock measures with both amyloid and tau PET imaging markers; the first study also showed significant association with the Preclinical Alzheimer's Cognitive Composite (24), while the second demonstrated the ability to further tailor the DCTclock algorithm to improve detection of Alzheimer's pathology (25). These results offer proof of principle of the broadened access of digital assessments, but also the greater accuracy in predictions through machine learning.

Linus Health improves health outcomes by providing convenient asynchronous cognitive, physical and behavioral testing that can be done in a familiar, less stressful environments than clinics. Linus Health can be deployed in the context of preventative care by establishing a cognitive health baseline in both primary and senior care facilities. This enables not only more frequent testing, but earlier testing that can potentially detect the beginnings of cognitive decline. Linus Health is currently being tested in 2 senior care facilities with over 100 patients, 12 research studies involving over 2 million patients, and 3 primary health care systems involving over 12 million patients. In total, Linus Health is being deployed by over 20 organizations in over 24 countries.

## Mind the Gap: Digital Solutions to Extend Timely Specialist Insights Into Primary Care to Close the Growing Chasm Between Lifespan and Health Span

GNPT, the BBHI coaching app and Linus Health all illustrate the concept and transformative potential of ITA. ITA addresses five key aspects to enable person-centered, early detection and intervention, ultimately promoting preventative, precision brain health, expanded upon below.

### Transcending a “Pit-Stop” Model of Care and Enabling Continued, Equitable Care

**Figure 2:**
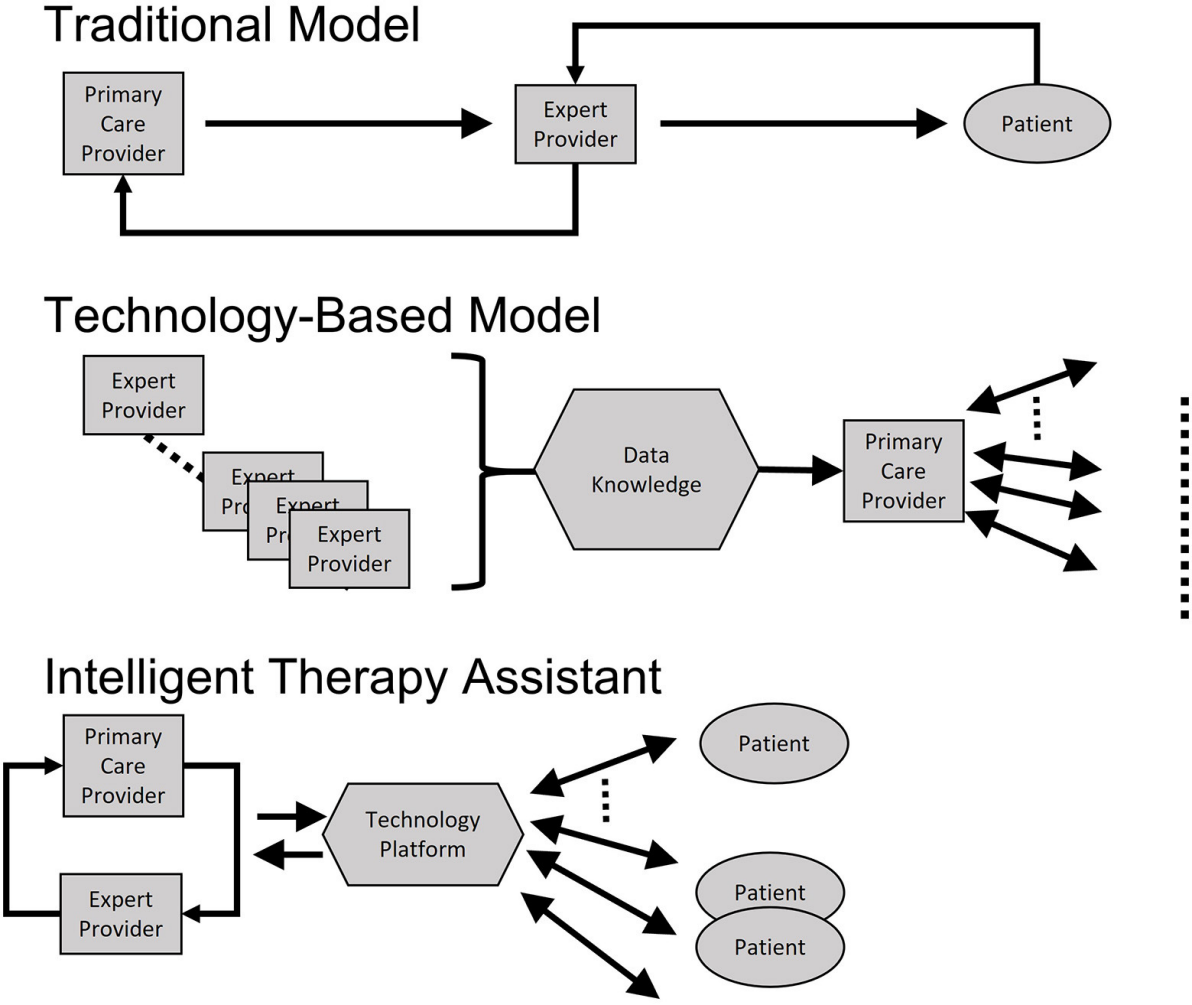
Schematic illustration of Intelligent Technology Therapy Assistant (ITA) compared with traditional care model and more standard technology-based approaches. Traditional approaches require independent visits to primary care and specialists (expert providers). Technology based approaches consolidate expert knowledge and disseminates via primary care. ITA promotes the integration of knowledge from expert providers and primary care, using technology to disseminate the integrated knowledge to many patients. Importantly, the use of artificial intelligence enables the optimization of such knowledge, with inputs from specialists, primary care clinicians, and empowered patients.

During a formula 1 race, a timely pit stop is critical. However, to truly increase the chances of winning the race, the pit stop must be efficient, comprehensive, and very short. To win, the car needs to be on the track and the technical support team needs to be in continued contact with the driver and monitoring the performance of the car in real time. Supporting brain health demands a similar approach: short but timely visits to the doctor or hospital stays, but ongoing monitoring and targeted, personalized interventions with the expert clinicians in the loop. Expert medical care needs to be delivered wherever the empowered patient wants to be, not expecting the patient to come to a clinic or hospital, but rather going to the patient, and enabling each individual to define their best vision of themselves and deliver them tools, recommendations and prescriptions to achieve that vision. The responsibility of multidisciplinary teams of experts is to support, educate, offer necessary tools and interventions, and join the patient and their loved ones along a journey of promotion of function even in the face of disease. ITA promotes the integration of knowledge from expert providers and primary care, using technology to disseminate the integrated knowledge to many patients. Importantly, the use of artificial intelligence enables the optimization of such knowledge, with inputs from specialists, primary care clinicians and empowered patients (Figure 2).

### Seamlessly Fitting Into the Expert Clinician's Workflow

Digital technologies need to be developed in collaboration with expert clinicians to successfully achieve the goal of extending their reach. Thus, they will ideally enable enrichment, from her or his validation, being able to modify or fine-tune the automatically obtained output with expert criteria. Specific knowledge of clinician's workflows to automate redundant processes [such as scanning a test into an electronic health record (EHR)] can maximize visit time for discussion and human connection. Another critical aspect is that new assessments be developed in ways to be integrated into existing workflows. While novel neuropsychological assessments can enable more frequent testing, a downside is the limitation in the ability to compare performance to established scoring systems that have been the basis for existing clinical decision frameworks. Ultimately, novel solutions will be fluid and customizable to different individual workflows and health systems.

### Contextualizing Information From Different Sources and Staying Up to Date

Recent advances in mobile and wearable technologies enable simultaneous collection of large amounts of data from completely different sources (neuropsychological, biomechanical, and EHR) at multiple time points. Although great volumes of data are being collected, meaningful analysis of such data often lags, which risks losing signals amidst the noise or oversampling, introducing bias in algorithm development. Thus, an important challenge is to understand, contextualize and apply this collective information and acquire a sufficiently robust dataset and implement analytical methods that can, among other things, ensure against AI bias. An additional consideration is the challenge in keeping digital solutions abreast of the wealth of new knowledge emerging daily. Successful digital solutions will therefore need to combine human expert knowledge with machine learning to develop AI-based algorithms that are constantly optimized in their predictive and prescriptive capacities.

### Business Solutions That Support AI-Based Digital Health Solutions

An important challenge is the need for appropriate payment models that vary in their flexibility in accommodating global digital solutions and regulatory compliance frameworks that were generally built for static pharmaceutical therapies and binary diagnostics (for instance billing practices that support predictive modeling algorithms, cloud services for large amounts of data, safeguarding data privacy and security, etc). The complexities of dementia-related impairments require that assessments and interventions must be adaptive to adequately understand and treat such a complex neurophysiological system that is both generalizable and specific to the individual, which makes software an ideal medium to practically broker measurements, understandings, and customized recommendations at scale. Healthcare must embrace and expand its adoption of enterprise software as a service (SaaS) business models and solutions to scale expertise, enable customization without compromise, and drive down costs while expanding access to an uplifted standard of care.

Digital health tools are part of the solution to finding effective ways to manage the health and social challenges posed by the growing proportion of people in the world at risk for age-related cognitive decline, and support clinicians, patients, families, and caregivers in financially feasible, scalable and sustainable manners.

## Data Availability Statement

The original contributions presented in the study are included in the article/supplementary material, further inquiries can be directed to the corresponding author/s.

## Author Contributions

JG-O, JS-S, ER, GC, ÁP-L, DB, WS-M, EG, JT-M, and DB-F participated in the study concept, manuscript preparation, and revision. All authors contributed to the article and approved the submitted version.

## Funding

This research leading to the reported results is funded by grants from la Caixa Foundation (grant agreement no. LCF/PR/PR16/11110004), Institut Guttmann and Fundació Abertis. DB-F was funded by the Spanish Ministry of Science, Innovation, Universities (RTI2018-095181-B-C21), and an ICREA Academia 2019 award research grants. JT-M was partly supported by INNOBRAIN (COMRDI15-1-0017).

## Conflict of Interest

JG-O, ER, WS-M, DB, ÁP-L were employed by the company Linus Health. The remaining authors declare that the research was conducted in the absence of any commercial or financial relationships that could be construed as a potential conflict of interest. The authors declare that this study received funding from the following funding sources: la Caixa Foundation (grant agreement no. LCF/PR/PR16/11110004), Institut Guttmann and Fundació Abertis, the Spanish Ministry of Science, Innovation, Universities (RTI2018-095181-B-C21), ICREA Academia 2019 award research grants and INNOBRAIN (COMRDI15-1-0017). The funder was not involved in the study design, collection, analysis, interpretation of data, the writing of this article or the decision to submit it for publication.

## Publisher's Note

All claims expressed in this article are solely those of the authors and do not necessarily represent those of their affiliated organizations, or those of the publisher, the editors and the reviewers. Any product that may be evaluated in this article, or claim that may be made by its manufacturer, is not guaranteed or endorsed by the publisher.
